# ‘So easy to destroy, so hard to build’: understanding impacts of federal rule changes on the Title X program, 2018-2024

**DOI:** 10.1093/haschl/qxag235

**Published:** 2026-09-28

**Authors:** Rebecca G Simmons, Jami Baayd, Caitlin Quade, Jessica N Sanders, Audrey Widner, Alexandra Gero, Sarah Compton

**Affiliations:** Division of Family Planning, Department of Obstetrics & Gynecology, School of Medicine, University of Utah, Salt Lake City, UT 84112, United States; Division of Family Planning, Department of Obstetrics & Gynecology, School of Medicine, University of Utah, Salt Lake City, UT 84112, United States; Division of Family Planning, Department of Obstetrics & Gynecology, School of Medicine, University of Utah, Salt Lake City, UT 84112, United States; Division of Family Planning, Department of Obstetrics & Gynecology, School of Medicine, University of Utah, Salt Lake City, UT 84112, United States; Department of Obstetrics & Gynecology, University of Michigan Medical School, Ann Arbor, MI 48109, United States; Division of Family Planning, Department of Obstetrics & Gynecology, School of Medicine, University of Utah, Salt Lake City, UT 84112, United States; Department of Obstetrics & Gynecology, University of Michigan Medical School, Ann Arbor, MI 48109, United States

**Keywords:** contraception, public policy, Title X, family planning

## Abstract

**Introduction:**

Title X is the only federal program that directly funds family planning care. Recent Rule Changes have resulted in back-and-forth shifts to the program. Our goal was to understand how the federal Title X Rule Changes have impacted grantees and their perceptions of future program participation.

**Methods:**

In 2024, we conducted qualitative interviews with 25 grantees and national partners and held listening session focus groups with 37 grantees and subrecipients.

**Results:**

We identified 5 themes: (1) Title X is seen as both programmatically burdensome and essential; (2) the 2019 Rule Change demonstrated how grantee organizational and state contexts impact their responses and decision-making; (3) external environmental factors created additional systems stressors; (4) the 2021 Rule Change resulted in return to the network but reintegration/ readjustment were taxing; and (5) existing grantee differences still impact capacity for future participation.

**Conclusions:**

We found that while Title X is viewed by grantees as a vital component of the reproductive health safety net, Rule Changes significantly increase the burden (regardless of program participation) of service delivery. Importantly for policymakers, subsequent recovery after program restoration is nonlinear and highly dependent on grantee characteristics and the local environments in which they operate.

## Teaser

Title X is the backbone of the federal family planning safety net, but what happens when the program itself becomes a source of instability? In 2024, we conducted qualitative interviews and listening sessions with over 60 Title X grantees, subrecipients, and national partners to understand how back-and-forth federal Rule Changes have reshaped the program from the inside. We found that all grantees, regardless of whether they remained within the network, experienced burdensome disruption with Rule Changes and that recovery from policy disruption is neither quick nor uniform. We noted that the heterogeneous nature of Title X grantees contains significant differences in grantee capacity and local context that differentially shapes who can sustain participation in this program. As Title X remains a politically charged federal program, our research offers a grounded practitioner-level view of what federal Rule Changes actually cost organizations on the front lines of implementation and what policymakers need to understand for future policy shifts.

## Introduction

Title X of the Public Health Service Act established the National Family Planning Program within the U.S. Department of Health and Human Services (HHS) in 1970.^1^ Signed by Republican president Richard Nixon, it received unanimous support from the Senate and bipartisan support from the House. After Title X's passage in 1970, court decisions and legislative action broadened the program beyond contraception and testing and treatment for sexually transmitted diseases and enabled more Americans to access additional reproductive health services, such as infertility screening.^2^ Yet, as time has passed, the program's existence and mission have become increasingly politicized and partisan. Specifically, its requirements to provide adolescent services without parental notice or consent^3^ and its inclusion of abortion providers (including programmatic requirements to provide abortion referrals to those desiring them^4^) have proven divisive, though the program notably prohibits funding for abortion services. As a result, Title X has remained level-funded at approximately $286.5 million since 2014,^5^ has faced dozens of federal lawsuits,^6,7^ and has undergone dramatic programmatic shifts with changing administrations.

One of the most seismic shifts for the program came with the first Trump Administration's 2019 Rule Change, which created strict restrictions around funding for organizations that provide abortion care and introduced several additional programmatic changes, including shifts in nondirective pregnancy counseling and referrals, physical and financial separations from abortion services, changes to contraceptive method requirements, and shifts in service delivery requirements for minors, among others (see Table 1). In response to the 2019 Rule Change, many entities withdrew from the program, including all Planned Parenthood affiliates (One Planned Parenthood affiliate remained within the program as a subrecipient after the 2019 Rule Change). Six states ultimately lost all Title X funding, and 24 states lost partial funding, reducing network capacity by 46%.^8^ Assessments of impact showed increases in the prevalence of contraception deserts, with a disproportionate impact on low-income populations and populations of color.^9^ In early 2021, the Biden administration proposed another rule change, reversing many, but not all, of the 2019 Rule Change requirements (Table 1). The Biden administration sought to bolster the network with additional short-term federal funding. This funding was provided through COVID-19 federal relief budgets, for “dire family planning needs” ($6.6 million) and for telehealth expansion ($16.3 million), amounting to an additional $23 million in support.^10^ Yet even with additional funding and support, 2023 Family Planning Annual Report (FPAR) data showed the network had not recovered. Client volume dropped from approximately 4.1 million distinct clients in 2018 to 1.5 million in 2020 and had still not reached the 3.0 million mark by end of 2023, 2 years after the Biden administration reversed the 2019 Rule Change.^11^

**Table 1.** Title X regulatory comparison table.

Domain	Pre-2019	2019 Rule Change (effective Aug. 2019)	2021 Rule Change (effective Nov. 2021)
**Governing Regulation**	2000 regulations (42 CFR Part 59); in place ∼2000-2019; last substantially updated prior to 2016 federal administration.	New final rule: “Compliance with Statutory Program Integrity Requirements” (84 FR 7714, Mar. 4, 2019); effective Aug. 19, 2019; revokes prior guidance	New final rule: “Ensuring Access to Equitable, Affordable, Client-Centered, Quality Family Planning Services” (86 FR 56144, Oct. 7, 2021); effective Nov. 8, 2021; revokes 2019 Rule in its entirety
**Abortion Counseling & Referral**	Required nondirective pregnancy counseling and referral on 3 options: (1) prenatal care/delivery; (2) infant care, foster care, or adoption; and (3) pregnancy termination/abortion, upon client request. (42 CFR §59.5(a)(5), 2000 rule)	Nondirective pregnancy counseling permitted, not required. Must be conducted by a physician/APP. Prohibited referral for abortion as “in most instances when referral is provided for abortion, that referral necessarily treats abortion as a method of family planning.” Any referral lists of providers could not identify which perform abortions. (42 CFR §59.14; 84 FR 7714 at 7748-7750)	Required nondirective counseling and referral on all 3 options (prenatal care/delivery; infant/foster/adoption care; pregnancy termination/abortion) at patient's request. Removed physician/APP-only restrictions on abortion counseling. Conscience protections preserved for objecting providers (footnote 2, 42 CFR §59.5). (42 CFR §59.5(a)(5); 86 FR 56144 at 56183–56188)
**Physical & Financial Separation from Abortion Services**	Abortion activities must be “separate and distinct” from Title X project activities. Shared facility, staff, waiting room, and records permissible if Title X and non-Title X activities are distinguishable. (42 CFR §59.9, 2000 rule; 65 Fed. Reg. 41276)	Strict physical and financial separation is required. “Objective integrity and independence” test. Financial separation is insufficient. Co-location for Title X and abortion services is effectively prohibited. (42 CFR §59.15; 84 FR 7714 at 7757-7760)	Strict separation requirement repealed. Reverted to pre-2019 “separate and distinct” standard. Co-location permissible again. (42 CFR §59.9; 86 FR 56144, Section VI, §59.9; preamble at 56198–56200)
**Contraceptive Method Requirements**	Required “broad range of acceptable and effective medically approved family planning methods” including natural family planning methods, infertility services, and adolescent-friendly services. Single-method or limited-method participation permitted as long as the full project collectively covers a broad range of methods. (42 CFR §59.5(a)(1), 2000 rule)	Removed “medically approved” language. Single-method or limited-method participation permitted as long as the full project collectively covers a broad range of methods. (42 CFR §59.5(a)(1); 84 FR 7714 at 7726-7730)	“Medically approved” language reinstated. Sites not offering a broad range must provide prescription or referral for client's method of choice. Telehealth explicitly authorized. (42 CFR §59.5(a)(1); 86 FR 56144 at 56170–56172)
**Services for Minors/Confidentiality**	Confidential services to minors; family participation encouraged per statute and Congressional mandate but could not override minor's wishes; discounts based on minor's own income for unemancipated minors seeking confidential services. (42 CFR §59.2; 42 CFR §59.11)	Added documentation requirements — minor medical records must indicate sexual partner ages; projects must document family participation actions or reasons it was not encouraged. Added screening for victimization of minors with STI/pregnancy/abuse. (42 CFR §59.17; 84 FR 7714 at 7755-7757)	Restored pre-2019 standards and added language that explicitly prohibits requiring parental consent or notifying parent/guardian when minor requests or receives services. 2019 documentation requirements revoked. (42 CFR §59.5(a)(14); 86 FR 56144 at 56190–56194)
**Antidiscrimination & Nondiscrimination**	Standard federal nondiscrimination requirements; no expanded “sex” definition	Conscience protections for providers strengthened. No new inclusive or antidiscrimination language added. Removed requirement for providers to counsel on/refer for abortion as conscience protection measure. (84 FR 7714 at 7741-7745)	Added expanded “sex” definition inclusive of sexual orientation, gender identity, and sex characteristics per ACA §1557. Services must be gender-inclusive; “clients” substituted for gendered language. Subrecipient nondiscrimination provision added (42 CFR §59.5(a)(18)). (86 FR 56144 at 56177–56183)
**Sliding Fee Scale/Client Eligibility**	Free at ≤100% FPL; sliding scale 100%-250% FPL; full cost above 250% FPL. Project directors had discretion (“good reasons”) to provide free/discounted services beyond guidelines. (42 CFR §59.5(a)(7), 2000 rule)	“Good reasons” director discretion provision eliminated. Maintained free at ≤100% FPL; sliding scale 100%-250% FPL; full cost above 250% FPL. (42 CFR §59.5(a)(7); 84 FR 7714 at 7750-7751)	Maintained 3-tier fee structure. “Good reasons” discretion provision not fully restored but fee structure unchanged. (42 CFR §59.5(a)(7); 86 FR 56144 at 56188)
**Funding Level and Grant Components**	∼$286 million/yr (FY2014-2018); 3-year grant cycles (FPAR 2018; OPA Annual Reports)	Appropriation unchanged (∼$286 million); 3-year grant cycle	Maintained $286.5 million/yr. $50 million 1-time ARPA supplemental (P.L. 117-2, §2605). 2022 NOFOs issued new 5-year grants. (FPAR 2023; HHS ARPA announcement)

42 CFR 59: Title 42 (Public Health), Code of Federal Regulations, Part 59 (Family Planning). ACA, Affordable Care Act; APP, Advanced Practice Provider; ARPA, American Rescue Plan, CFR, Code of Federal Regulations; FPL, Federal Poverty Level; FPAR, Family Planning Annual Report; FR, Federal Register; HHS, U.S. Department of Health & Human Services; NOFO, Notice of Funding Opportunity; OPA, Office of Population Affairs; STI, sexually transmitted infection.

Yet numbers alone cannot fully capture the impact of the 2019 Rule Change on the Title X network. Quantitative data reflect what changed, but not how grantees experienced those changes or how local context shaped their ability to adapt. This study draws on qualitative data to examine grantees' experiences navigating both the 2019 and 2021 Rule Changes and how those experiences have informed their plans for future Title X participation.

## Data and methods

### Theoretical framework

We designed this study as an explanatory sequential mixed methods project. Explanatory sequential mixed methods design utilizes quantitative data to test a hypothesis, followed by qualitative data collection to interpret and clarify findings.^12^ As our study intended to examine not only the impact of regulation change on service delivery outcomes, but also how states and clinics responded to the changes, this approach allowed us to examine these factors methodically.

To achieve our aims, we first conducted the quantitative strand of our study, the results of which are published elsewhere, examining how changes in Title X regulations affected patterns of contraceptive and other sexual and reproductive health (SRH) service provision.^13^ Briefly, we found that the level of Title X capacity reduction in 2019 had additional impact on certain outcomes, such as types of contraceptive methods used and sexually transmitted infection testing, but less impact on others, such as total clients served. While 2019 capacity loss was a significant predictor of specific outcomes, it did not significantly predict the pattern of non-recovery observed after the 2021 Rule Change, prompting our qualitative inquiry into unmeasured contributors to this gap. Findings from these analyses were presented in a 3-day meeting (October 2023), which then led our team to identify several key clarifying questions to explore through qualitative research. These questions were broken down into 4 specific areas: (1) pre-2019 Rule Change actions/behaviors; (2) experiences of 2019 Rule Change implementation; (3) 2021 Rule Change actions/behaviors and recovery; and (4) expectations for the future. This study received Institutional Review Board Approval from the participating institution (IRB #00159744).

### Participant selection

We had a priori determined to interview representatives from organizations who had received Title X grants (referred to as “grantees”). Typically, Title X grantees have specific administrators or directors who support both the Title X application and its administration across their grantee network, which typically includes subrecipients and/or service sites. Our quantitative findings suggested that capacity loss was only one factor impacting outcomes, but we suspected additional variables, such as state political environment and grantee type (eg, a state health department compared to a healthcare nonprofit organization) also likely impacted response to federal rule changes. Ultimately, we determined our participants should include representation across the following characteristics: (1) level of capacity loss in 2019 Rule Change (none/some/total loss); (2) geographic diversity (ensuring regional representation across the United States); (3) grantee type (state health department only; only 501c(3) nonprofit organizations; a mix of both); and (4) the political leaning of the state (liberal, conservative, mixed), as we assessed that state policies likely impacted grantee capacity.

Our quantitative findings indicated that state and grantee-specific contexts likely shaped outcomes, but we also recognized that large national entities could offer important perspective on how federal rule changes played out across their broader networks. For example, the decision for all Planned Parenthood affiliates to withdraw from the Title X program was largely influenced by Planned Parenthood Federation of America, rather than being decided by individual affiliates. Similarly, the National Family Planning and Reproductive Health Association (NFPRHA) serves as the membership organization for Title X-funded entities, providing technical and operations support to members, including representation in litigation challenging the 2019 Rule Change (*National Family Planning & Reproductive Health Association, et al. v. Azar, et al*; *State of Washington v. Azar, et al.*).^6^ Given these important perspectives, we ultimately determined we would conduct in-depth interviews with Title X grantees from 9 states and 4 national entities (NFPRHA, Planned Parenthood Federation of America, Office of Population Affairs [OPA], and National Association of Community Health Centers [NACHC]). In settings with more than one grantee, we attempted to interview each grantee. If, in the process of an interview, a grantee identified subrecipients that experienced relevant differences in the grant implementation process, we also reached out to the identified subrecipients for follow-up interviews.

### Data collection

Interviews were conducted between December 2023 and April 2024. One or 2 team members (JB, CQ, JNS, and RGS) attended each of the interviews, which were conducted electronically using Microsoft Teams or Zoom, depending on participant preference. Interviews took approximately 60 minutes. Appendix S1 provides an overview of our generalized interview guide, which was modified slightly for national partner organizations. Participants were offered $100 as compensation for their time in providing these interviews, though not all elected to receive compensation. All interviews were recorded and transcribed.

Additionally, given the size and heterogeneity of the network, we felt the need to triangulate initial qualitative themes and explanatory variables following our in-depth interviews.^14,15^ To achieve this, we recruited Title X grantees/subgrantees attending the NFPRHA conference in May 2024 to participate in a 90-minute focus group session. Interested individuals signed up for the session and were provided with breakfast. We separated participants into focus groups by loss levels during the 2019 Rule Change period. Some grantees who had previously participated in interviews also attended this session. As the purpose of these focus groups was confirmatory, we took field notes for each focus group but did not record or transcribe sessions. All members of the study team participated in focus group facilitation.

### Analyses

Transcribed data were uploaded to MAXQDA v. 2024. Five members of the team participated in data coding (JB, CQ, JNS, RGS, and AW). Our team group coded 3 transcripts—2 grantee interviews and 1 national partner interview, to develop an initial codebook. Using a consensus coding approach,^16^ team members coded the remaining transcripts separately and met biweekly to discuss potential additions to the codebook and obtain consensus around emerging coding questions. Any disagreements were resolved by group discussion. Upon completion of initial coding, all team members met for 2 coding synthesis meetings to discuss themes and data saturation and identify any areas of missingness. Participants were provided the opportunity to review and approve all findings disseminated from this data collection, including those presented in this paper.

## Results

We conducted 18 in-depth interviews with a total of 25 people across 9 states (see Table 2) and 4 national entities. Additionally, 39 Title X grantees representing programs from 27 states attended the focus group listening session. Overarching themes are reported below; Table 3 presents illustrative quotations for each theme.

**Table 2.** Title X grantees identified for in-depth interviews.

State	Level of capacity loss in 2019 Rule Change	Geographic diversity (Title × Region)	Grantee type(s) in the state and number of interviews conducted	State political leaning (2024 Cook Partisan Voting Index)
Texas	No loss	6	Nongovernmental 501c(3) (*n* = 2)	Republican (R + 6)
Tennessee	No loss	4	Non-state 501c(3) (*n* = 2)	Republican (R + 14)
Nevada	No loss	9	State/county and non-state grantees (*n* = 1)^a^	Republican (R + 1)
Maryland	Partial loss	3	State (*n* = 1 + 1 subrecipient)	Democrat (D + 15)
Michigan	Partial loss	5	State (*n* = 1)	Even
Missouri	Partial loss	7	Non-state 501c(3) (*n* = 1 + 1 subrecipient)	Republican (R + 9)
Washington	Total loss	10	State (*n* = 1)	Democrat (D + 10)
Maine	Total loss	1	Non-state 501c(3) (*n* = 1)	Democrat (D + 4)
Utah	Total loss	8	Non-state 501c(3) (*n* = 3)^b^	Republican (R + 11)

^a^Nevada had 5 Title X grantees at the time of recruitment. We attempted to recruit all grantees, but only successfully recruited 1 grantee.

^b^Utah's Title X leadership had completely changed during the study, where no leadership from the 2019-2021 Rule Change period currently worked at the organization. As such, we discussed the 2019 Rule Change and prior years with a former staff member and the 2021 Rule Change time period and beyond with 2 members of current staff.

**Table 3.** Example participant quotes from thematic findings around the impact of federal rule changes on the Title X program.

Theme	Quotes
Theme 1: Even before the rule changes, the Title X program was seen as both burdensome and essential	*“I mean, Title X is a really complicated program, right? For people that have to run a Title X grant in their state, there is so much to the Title X program. I mean, you have to know how to do a budget and get funding out through your health department or your agency to all these different subs. You also need to know how to bill all the insurances and Medicaid and how to do the electronic health records and how to collect data. And you need to know all the clinical service delivery and quality standards. There is so much to Title X on a good day. And then you add to that the staff turnover, the loss of institutional memory, and the just sheer exhaustion of working in this field…So, it's just complicated on a good day.”—[National Organization 3]* “*I mean, so 340B is absolutely fundamental to health center financial stability. Without the 340B program, health centers' doors would close.”—*[*National Organization 2]* *“I think one of the things that keeps coming up is it's not that much money. You’re like, “But i’'s not about the money. It's this network piece. It's this mission. It's all the other benefits.”*—*[Nonprofit grantee 4]*
Theme 2: The 2019 Rule Change and its fallout underscored existing disparities among grantees that fundamentally affected their decision-making	*“We worked really hard to say no one had a good choice here. You’re either forced to forego the federal money and try to make the best you can with limited resources, or you’re forced to undermine what you believe, and we believe to be the quality of care. You’re forced to threaten the credibility and quality relationship that you’ve built with your patients because you’re holding on in order to be there for people who need your care.”—[National Organization 1]* *And so, [State]'s always been, when it comes to access to reproductive health, I mean, specifically abortion, but it runs the gamut, has always been hostile. And there has been a chipping away at people's rights since 1973. And so there's always been different hoops and hurdles, and sort of just an environment that people have had to sort of live with and operate within. And one thing I’ve always said even with the Trump rules, our folks are used to sort of jumping through lots of hoops, have bended themselves into pretzels to get people the care that they need. So that didn’t feel unusual.—[Nonprofit grantee 2]* *“We had several meetings with the Governor's office. Aside from [State] being pro-sexual and reproductive health and wanting these services out there, there is a law on our books that says that they need to provide contraceptive services to people who can’t afford it.—[State grantee 1]* *The other thing that I would say was a big part of the decision making was that most of the states with supportive administrations were hearing from their governor's offices or departments of health that they were going to continue to receive state-based family planning dollars as a trade-off if the states pulled out of the Title X program. And the states were really out front working with us, like in some of the litigation and in the strategy to make it very clear that they were going to pull out of the program too. That made a big difference in determining the path forward, I think, for folks.—[National Organization 4]*
Theme 3: Regardless of whether grantees remained in the program, 2019-2021 was a particularly challenging time for organizations	'*So, at the same time that we were raising private funds to replace the federal dollars that we rejected, we also put in a bill at the state level to basically replace the federal money. If legislature hadn’t closed because of the pandemic, I think we might have gotten funding. We probably would have gotten funding if the pandemic hadn’t happened and the legislature had not shut down.”—[Nonprofit grantee 4]* *“I mean, I think the biggest impact was the time suck. I don’t want to say that's the biggest impact, but I think that's what gets lost in so much of all of this. Yes, the impact that it has on people, but the impact it has on agencies and individuals. So now, if you have to devote your entire time to figure this out, engaging with conversations amongst your team, individual conversations with subrecipients, walking them through stuff, all those things, that is time you’re no longer spending, again, on the innovative solution, on trying not just to survive, but to really meet community need. So it's the time, energy and the mental toll that it has in terms of all of that.”—[Nonprofit grantee 5]* *“I mean, at the same time that this was happening, the federal office was also undergoing immense change. The staff that worked with grantees used to be run out of the regional offices. And when OAH and OPA merged together, all the staff from the regional offices were pulled to the national office. And the program was run centrally, which practically meant all the staff from the regional offices left OPA. So, we also at this time, as this was all happening, had to completely hire a brand-new staff and then train all new staff on everything Title X.”—[National Organization 3]* *But there was a whole period where we were not only implementing our program and training our subrecipients, we were training OPA on their program and what they really needed to know and what they didn’t need to know.* *- [Nonprofit grantee 1]* *COVID, like everything, really, really affected our subrecipients. Loss of staff, staff being out sick, trying to switch over to a telehealth model when they really didn’t have a telehealth model, trying to do that quickly. I’m really proud of our subrecipients, but we’re still feeling the effects of the staff, especially providers and MAs. Because they can get such higher pay in other areas.—[State grantee 1]* *“And I think especially those health departments that were trying to join the Title X network, it was particularly hard for them because they were swamped with COVID activities and doing immunizations and testing. Everybody was just so swamped that… And we couldn’t visit and work with them, so it was very difficult to… It really made it much harder to try to start a program or get things going.”—[State grantee 2]*
Theme 4: Many grantees who had left the program opted to come back after the 2021 Rule Change, but reintegration and shifting policies also had challenges.	*“And I think that that's where our affiliate was very challenged in getting back into the grant program is because of how fragmented the leadership had become and the infrastructure had become by the time they got back into it. I don’t think they had the bandwidth to really dive deep and understand the changes that had happened in the grant with the renewal and really put together a broad implementation plan for those changes and all the new staff. Because some of the leadership had been there throughout it all. I’m not sure that they appreciated that most of the people on the ground were new and this was new to them and that they would need full training from the ground up again. I think it was just kind of, “And we’re back,” and there wasn’t that careful thought process of like, “Who do we need to train? How do we need to train? How do we need to separate funds again? How do we put that clarity that we once had around sliding scales and eligibility?” And our finance group didn’t have the training in how to separate it or what it meant. So, the regulatory compliance part of it just did not come along with it because everything fell apart while it was gone.”—[Nonprofit grantee 3]* *'So, I think for every individual that pulled out, there was a different reason that we were just not able to address. And then I think some of the recovery has been just funding because it's been so flat funded. And then when sites left, [the grantee] got on it and onboarded new sites, and so that just kind of like squeezed out the ability for the other sites to join. We also haven’t had a competitive cycle, I don’t think, since that happened. –[Nonprofit grantee 1]* “*The aftereffects of this* [rule change, then changing back] *have been people still are not getting the right information. No matter how hard we try, providers are scared. They don’t want to do the wrong thing because they don’t want to risk the program, and then our patients are the ones that are suffering and not getting the information that they need because of this. –[Nonprofit grantee 6]* *“But I think a huge consequence of these swings is staffing, is workforce development. You’ve got people who know a lot and you’ve got people who’ve been in the program for a year or two. It's really hard. Thank goodness we have a project officer who knows the program but there's nobody… I mean, it's so difficult as a manager to say, “Oh, yeah, sign on for this program. You're really passionate about reproductive health.” We didn’t know we had a budget until last week. We still don’t have our full budget. Contracts start next week. You don’t want to completely alarm people and this is real life shit.” –[Nonprofit grantee 2]*
Theme 5: Existing disparities between grantees impact how they consider future participation in the program	*“And so we are an academic institution. We function off of gold standards, clinical guidelines, evidence, research, all of these things, and some of these things are falling outside of that, and do we want to continue to participate in a program that doesn’t support science? Was kind of the other kind of question that we had to really grapple with. You know, did the benefit of having the program outweigh the risk of providing care outside of science? –[Nonprofit grantee 6]* *“What we’ve told our staff, and it seems weird, but this is as good as it gets for Title X. No one on our staff except for [PERSON] and I worked Title X under the Trump years. Trying to prepare everyone for it's going to get harder and it's going to get harder for us to do the right thing. We’re going to do the right thing. What happens if the feds say you can’t provide emergency contraception as part of your contracts? Then what do we do? How far do we hold the line?’* *—[Nonprofit grantee 4]* *‘I’m kind of going, “Oh my god, I don’t want to think about that.” So right now it's quiet, but I think this time…I learned my lesson last time. I resisted scenario planning for quite a while, thinking that it was going to take a long time for the rule to change. But now I know better. So I think we will start some scenario planning to have ready for if that occurs because otherwise its just a scramble to get everything together.”—[State grantee 1]* *“I am not sure. I think we’re in a different situation. The healthcare landscape in general is so much more challenging than it was even four or five years ago because…like jobs, the inflation challenges, just all of these external challenges that are aside from the significant problems that we have in sexual and reproductive health. I don’t like talking about Title X as a bottom-line program, but I think that the bottom-line implications are different than when we were engaging with this six years ago. In some ways, I think it's more complex than it was before, given the other variables.” [National Organization 4]*

**Theme 1: Prior to the 2019 Rule Change, participation in the Title X program was already challenging for grantees. Grantees participate despite burdensome administrative requirements because the program is seen as essential.** No one we spoke with considered the Title X program easy to participate in, even without shifts in requirements. Participants discussed data requirements, grant applications, and reporting as fundamentally more challenging than with other funding streams. Yet participants felt the program was an essential component of the family planning safety net, not only for financial support, but also for the key aspects of Title X, including access to 340B drug pricing, protections for confidential adolescent services, and connection to the larger network of family planning care.

**Theme 2: The 2019 Rule Change and its fallout underscored existing differences between grantees related to both their state policy and organizational funding environments.** In the aftermath of the 2019 Rule Change, grantees had to determine whether they were willing and able to participate in the redefined program or whether they needed to withdraw. Certain organizations did not feel that they could stay, ideologically. Other organizations faced challenges in maintaining compliance with different programs. For example, Planned Parenthood Federation of America found that compliance with the 2019 Rule Change would directly contradict the federation's Medical Standards and Guidelines,^17^ thus meaning affiliates could not maintain federation accreditation *and* Title X status. As a result, most affiliates were faced with a decision between accreditation status and Title X participation. For other grantees, existing state policies around abortion referrals and care made compliance with the 2019 federal Rule Change impossible. At the same time, in states with more restrictive reproductive health contexts, Title X represented one of the only dedicated family planning funding streams, leaving many sites feeling compelled to continue participation to ensure continuity of care for their populations. Decisions around this were challenging, regardless of the direction.

**Theme 3: Regardless of whether grantees remained within the Title X program during the 2019-2021 period, this timeframe was a particularly challenging time to provide care.** For those who left the network, there was a scramble to secure new funding and bolster support for lost services. For those who continued to receive Title X funds, determining how to implement the rule change while maintaining high-quality care took significant resources. Compounding these pressures, the OPA was restructured during this period, introducing further uncertainty for grantees navigating the transition. Finally, the COVID-19 pandemic disrupted operations during this time, with staff turnover emerging as a particularly widespread challenge.

**Theme 4: Many grantees that left the program opted to come back after the 2021 Rule Change, but reintegration came with its own set of challenges. For those who had remained, re-shifting policies and practices proved similarly demanding.** Some subrecipients that left the program during the 2019 Rule Change had been replaced by other organizations, and as such, even though entities were interested in returning after the 2021 Rule Change, many grantees did not have sufficient funding for those returning providers. Other former recipients had not maintained the rigorous reporting requirements of the program and found that staff turnover and loss of Title X expertise made reintegration into the program very challenging. Among ongoing recipients, the 2021 Rule Change required another round of policy and practice updates, while grantees also addressed staff fears and misinformation about what activities were permissible under the new rule.

**Theme 5: Existing differences between grantees impact how they consider future participation in the program.** Most participants expected future instability in the Title X program, anticipating that it would become similar to the global Mexico City Policy (MCP). The MCP experiences successive administrations immediately shifting rules and expectations around key programmatic components, creating cycles of disruption for implementing organizations.^18^ Grantees trying to remain in the program discussed grappling with the potential of future rule changes and challenges to providing evidence-based care. Grantees that had previously left the program discussed whether it was worthwhile to engage in a perpetual back-and-forth cycle, given the administrative and staff burden. Many felt it may not be. The availability of other funding sources and concerns about future restrictions to the program (eg, parental consent requirements) featured prominently in grantees' assessments of their future ability to participate.

## Discussion

The 2019 and 2021 Rule Changes have introduced a sort of “natural” experiment into the national SRH safety net. What happens to a network when seismic programmatic shifts are introduced and then rescinded? To date, several studies have explored the impacts of this experiment on specific areas of access. Studies exploring the impact on clients found that the loss of funding increased geographic barriers to access^9,19^ and resulted in changes to service delivery, including a reduction in available contraceptive methods.^20^ Research examining impacts on clinical providers reported effects on morale, challenges to restructuring counseling, increased clinical costs, and uncertainty about network return.^21,22^ Our qualitative findings, exploring impacts at the grantee level, mirror many of those identified at direct service levels. Grantees reported similar challenges, with additional administrative and legal burdens around guidance interpretation and programmatic compliance. Many of those that withdrew faced additional burdens of fundraising and revising financial policies, such as shifting sliding fee scales and absorbing costs previously offset by 340B drug pricing. However, several key distinctions exist.

We found significant differences in the impact of both the 2019 and 2021 Rule Changes, depending on the grantee. Unlike other federal programs administered by block grant, Title X is a competitive grant program wherein grantees demonstrate their capacity to provide high-quality care to populations in need of publicly funded family planning.^23^ The result is a heterogeneous grantee network operating within various state and organizational contexts that ultimately influence their capacity to respond to major shifts in federal policy. As such, understanding the impacts of federal program changes requires attention not only to funding losses, but to these broader contexts. For instance, in times of federal change, state governmental grantees may have lower programmatic flexibility than other types of grantees, but those that were compelled to withdraw from the program were also typically better resourced than other types of grantees to maintain service delivery outside federal funding—at least in the short term—due to supportive state investments in sexual and reproductive services. Additionally, nonprofit grantees can respond to federal funding restrictions in different ways, such as shifting to philanthropic and donor fundraising. Certain recipients, like many Planned Parenthood affiliates, have historically had significantly greater capacity to meet programmatic service demand,^24^ yet are also heavily influenced by the guidance of their national federation, which may constrain individual affiliate flexibility in responding locally to federal policy change. When considering the impacts of Title X Rule Changes on service delivery, both the type of grantee and existing state SRH policy context are critical factors in understanding outcomes.

Grantees that maintained Title X programmatic expertise, regardless of continued participation status, were better poised to respond to changes. Most participants agreed that administering the program is challenging even absent Rule Changes; the impact of expertise in supporting implementation during program shifts was significant. Those who lost expertise through leadership turnover or staff changes, whether due to the pandemic or program withdrawal, faced higher barriers to full program return. This deep knowledge and administrative expertise are particularly important because, in recent years, staff turnover and restructuring in the OPA has meant that federal staff overseeing service grants may have relatively little experience with administering Title X. More recently, there has been uncertainty about the future of the OPA entirely. Even though the office was created under the Title X statute, in 2025 HHS Secretary Kennedy proposed eliminating OPA along with many other HHS agencies. In addition, in October 2025, OPA staff were subject to a Reduction in Force (RIF)^14^ during the government shutdown. While federal jobs were restored as part of a bipartisan agreement to end the shutdown, no additional protections to OPA are in place. Lapses or cuts in federal staffing to oversee the program and provide direction to Title X grantees make on-the-ground programmatic expertise even more crucial.

Importantly, there were significant differences in how grantees considered future participation. Interviews were conducted when grantees were in Year 3 of a 5-year grant cycle. When asked to consider future participation in the program, many acknowledged that the program's politicization made it resemble the MCP wherein foreign aid is restricted among governmental and nongovernmental actors who provide information or services related to abortion. MCP has become a policy that oscillates under successive administrations, resulting in shocks to the aid community with each change.^25-27^ Most participants expected the Title X program to undergo additional rule changes moving forward with changing administrations. Participants noted that their considerations for staying in such unstable circumstances related largely to the healthcare needs of their clients. Many noted that in states with less generous SRH support, Title X remains one of the only options for funding access to low-cost SRH services, compelling many grantees to continue participation despite the increasing burden. Where state policy was more supportive (eg, states with Medicaid expansion, family planning state plan amendments, generous 1115 waivers, state-funded programs), grantees acknowledged that the costs of tumult might outweigh the benefits. Studies on the impact of MCP have similarly found that the back-and-forth nature of the policy's implementation has a chilling effect on organizational engagement.^28^ Yet, the challenges to Title X may not be limited to rule changes. Administrative or programmatic priorities can also impact the network, such as the Notice of Funding Opportunities (NOFO), which outlines grant eligibility and priorities for participation in the program. The ability of grantees to competitively score against other applicants and thus receive funding at all is directly tied to their ability to match programmatic goals to administrative priorities. Discretionary administrative grant enforcement can also impact existing grantees. For example, in 2025, 16 Title X grantees had Year 4 grant funding temporarily withheld, resulting in clinic closures and additional stressors for these sites.^29^ As grantees lose confidence in the stability of the Title X program, the strength of the overall network and its influence on critical elements of family planning care, such as standardized quality metrics, will likely be impacted disparately.

This study has some limitations. While we were able to interview many grantees, not all participants could speak to the full historical context of the Title X program, as some were newer in their positions. Where possible, we attempted to contact former staff for additional context, but it is possible that some themes remain underdeveloped in certain contexts. Our triangulation of interview data with field conversations among Title X grantees provided supplementary background and ultimately confirmed the main thematic elements we had identified. While we attempted to recruit across a wide swath of Title X settings, as mentioned, the program's heterogeneity means that additional considerations possibly remain uncaptured in our findings, particularly among grantees or organizations that are less engaged with the program.

## Conclusion

The Title X program has undergone fundamental shifts in the last decade. Our findings provide additional information to documented impacts, demonstrating that, like other politicized programs, grantees and service sites participating in Title X are significantly impacted by systemic shocks to the program. However, decisions about program participation are based on more localized factors, including their own capacity and flexibility to adapt, state contexts that hinder or support them in providing care, financial resources, and the needs of their patients, who remain centered in their decision-making. Future studies assessing the impacts of federal rule changes to the Title X program will benefit from incorporating these explanatory factors into their research. Policy makers, particularly state policy makers, should take note of our findings indicating the important role they can play in securing the SRH safety network during times of federal restriction.

## Supplementary Material

qxag235_Supplementary_Data
