# Supplementary material for: ‘So easy to destroy, so hard to build’: understanding impacts of federal rule changes on the Title X program, 2018-2024
Source: Health Aff Sch. 2026 Sep 28;4(9):qxag235. doi: 10.1093/haschl/qxag235 (PMC13617662; doi:10.1093/haschl/qxag235)
Supplement: Supplementary file 1 [file qxag235_supplementary_data.zip › Appendix Materials_20AUG2026.docx]

```
APPENDIX 1:
Title X Interview Guide
[Introductions interviewer team and interviewee]

First, thank you so much for taking the time to talk with us today. We’re really grateful for your support. As we mentioned in the recruitment email, we’ve received some funding from the Office of Population Affairs to study the impact of the 2019 Rule Change on sexual and reproductive health across the country. Our study involves interviewing people from nine states about their differing experiences with Title X, the 2019 Rule Change, and the aftermath of the Rule Change, including how Title X has been affected by other policies, such as the Dobbs decision. We expect that this interview will take about an hour.

Before we get started, do you have any questions about the study or this interview?

[Answer any questions]

Are you ok if we begin recording?

[Answer]

[START RECORDING]

Ok, to kick things off, why don’t we start by having you tell us a little bit about your role and how it intersects with the Title X program in [state].
	     Follow-up questions IF NEEDED
```

- How long have you been in this role?
- How long have you been working within the Title X program?

[Answer]

**Depending on how long the participant has been in the role, please select the question sections that apply to them.**

**PRE-GAG PERIOD**

We’re really interested in understanding how the Title X program was working in [STATE] prior to the 2019 Rule Change and how different organizations accounted for the rule change, once it became known that it was going to take effect in 2019.

Can you tell me a little bit about how you felt your Title X program was going prior to the 2019 Rule Change?

Follow-up questions IF NEEDED

- What would you say were the primary challenges the program was facing at the time?
- What types of things were happening either in the organization or in the state that were affecting the program?
- Who would you say was the primary population the Title X program was serving?
- What would you say were the needs of the Title X program prior to the Rule Change implementation?

```
DURING GAG RULE PERIOD
When it was formally announced in early 2019 that the Trump administration would be implementing the Rule Change, what you would say was the feeling or response by your organization?
	Follow-up questions IF NEEDED
```

- What conversations were had about the upcoming changes?
- What actions were taken ahead of the forthcoming rule?
- How did people in your organization make the determination about whether they were going to stay in the Title X program or leave?
- What types of funding or other revenue conversations were had regarding the upcoming ruling?

```
The Rule Change formally went into effect in August of 2019. What can you tell me about the immediate impacts of the ruling on your organization or state?
	Follow-up questions IF NEEDED
```

- What was the impact on clinical care?
- What was the impact on the provision of services (types of services offered)
- What was the impact on staffing/employment/retention?
- ***IF APPLICABLE - What, if any, differing funding streams were required to support your organization if it withdrew from Title X?

***IF APPLICABLE: The Rule Change noted that Title X grantees had until 2020 to complete the physical separation between Title X and abortion services. What, if anything, can you tell me about how this process went in your organization?

***IF APPLICABLE: Were there any challenges to implementing Rule Change requirements? (e.g., additional counseling requirements for adolescents, screening for sex trafficking, etc)

As the Rule Change went on, what can you tell me about the longer-term impacts on your organization?

Follow up questions IF NEEDED

- What were the strategies you took to maintain high quality care (e.g., for adolescents, for people seeking abortion services, for people in need of no-cost contraception)

```
What, if any, other effects did you see in other organizations or within your state?

What did your organization do to ensure that sexual and reproductive health was prioritized in your state during this time period?

The Rule Change also overlapped with the COVID-19 pandemic. Is there anything about the combination of those two events that you think is relevant to understanding Title X either then or now?

What, if any, other state policies or environmental challenges to providing sexual and reproductive healthcare were happening during this time?
	Follow up examples, IF NEEDED
```

- Medicaid/contraceptive coverage
- State policies around adolescents
- State policies around contraceptive access
- LGBTQ restrictive policies
- Abortion restrictions
- Outbreaks of sexually transmitted infections

Anything else you think we should know about your organization or state during the Rule Change period?

**POST GAG RULE**

In January of 2021, when the Biden Administration took over, they announced that they were rescinding the Rule Change. However, lawsuits and other challenges to the ruling meant that the actual ruling wasn’t rescinded until November of 2021.

What can you tell me about the conversations that were happening in your organization or state during this transition period?

How did your organization approach the transition?

- Clinical care
- Staffing
- Services offered
- Grant applications
- Funding

```
***IF APPLICABLE: What conversations did your organization have about reapplying for Title X funding?

***IF APPLICABLE: What challenges existed in re-entering the Title X program?
		Follow-up questions IF NEEDED
```

- Unexpected delays?
- Changes in expectations in the grant now vs. then?
- Changes in clinic care/services/staff
- Changes in state policies or other elements that impacted the program?

```
What has been your experience with the Title X program now?
	Follow up questions IF NEEDED
```

- Biggest challenges
- Biggest benefits

```
FUTURE Rule Changes

Similar to the 2019 Rule Change, there is relative consensus among policy experts that future Rule Changes are likely from now on, depending on the political party in office.

What conversations, if any, have you had with others (either in your organization or elsewhere) about the likelihood of a future Rule Changes?

What plans or steps have you taken, if any, to prepare for the event of a future Rule Changes?

What things would you do differently this time, compared to last time?

What type of support would be helpful to your organization in the event of another Rule Changes?

What advice would you give other states or organizations about what they can do to support sexual and reproductive health in the event of another Rule Changes?

What are the biggest concerns you have around the Title X program in the future?
	Follow-up examples IF NEEDED:
```

- Funding
- Rule Changes
- State environments around abortion or SRH
- Shifting clientele
- Shifting need for service types
- Insurance coverage
- Provider/staffing
- Reporting/data burdens

We’ve reached the end of our questions. Is there anything else you’d like to tell us before we end this conversation?

[ANSWER]

Thank you so much for participating today! We’ll be sending you your gift card information following this call. If you have any other questions or things you’d like to contribute, please don’t hesitate to reach out to us. We’re so thrilled to get to hear your experiences.
